# High Vimentin Expression Predicts a Poor Prognosis and Progression in Colorectal Cancer: A Study with Meta-Analysis and TCGA Database

**DOI:** 10.1155/2018/6387810

**Published:** 2018-05-31

**Authors:** Le Du, Jingchuan Li, Lei Lei, Hongjuan He, Erfei Chen, Jing Dong, Jin Yang

**Affiliations:** ^1^Key Laboratory of Resources Biology and Biotechnology in Western China, Ministry of Education, College of Life Science, Northwest University, Xi'an 710069, China; ^2^Institute of Preventive Genomic Medicine, Xi'an 710069, China; ^3^The Fourth Military Medical University, Xi'an 710032, China

## Abstract

The aim of this study was to evaluate the role of vimentin expression in the prognosis and progression of CRC. Meta-analysis was conducted to investigate the correlations between vimentin and prognosis and clinicopathological features in CRC. Literatures were searched by PubMed, Embase, ClinicalKey, CNKI, VIP, and WanFang databases. The Cancer Genome Atlas (TCGA) database was used to assess the association of vimentin expression with survival rate in CRC. Eleven reports with 1969 cases were included in the meta-analysis. The results showed that positive vimentin expression predicted a poor overall survival (OS) in the univariate analysis (HR: 2.087, 95%CI: 1.660-2.625) and multivariate analysis (HR: 1.633, 95%CI: 1.223-2.181). Vimentin overexpression also conferred worse disease-free survival (DFS) in the univariate analysis (HR: 2.069, 95%CI: 1.024-4.179) and multivariate analysis (HR: 2.802, 95%CI: 1.421-5.527). Moreover, upregulated vimentin is related to lymph node metastasis (OR: 2.288, 95%CI: 1.159-4.517), TNM stages (OR: 1.957, 95%CI: 1.333-2.873), and N stage (OR: 2.316, 95%CI: 1.482-3.620). Analysis of TCGA database indicated that elevated vimentin predicated a shorter OS (*p*=0.033). Our findings reveal that upregulated vimentin contributes to the progression and poor prognosis of CRC. Vimentin may be a prognostic biomarker and therapeutic target in patients with CRC.

## 1. Introduction

Colorectal cancer (CRC) is one of the most prevalent human malignancies and is considered as the fourth most common cause of cancer-related deaths worldwide [1, 2]. Although the overall incidence rate of CRC has declined, largely due to early clinical diagnosis and advanced therapies in developed countries, it is still very high in the East Asia [3]. Besides, the majority of patients with CRC suffer a poor clinical outcome, mainly because of unfavorable prognostic factors including distant/regional metastasis, local recurrence, and chemoresistance. Hence, increasing studies have focused on the molecular events related to these factors in CRC development, of which epithelial-mesenchymal transition (EMT) has received great attention in clinical research [4]. EMT is a dynamic process in which cells lose epithelial characteristics and acquire mesenchymal properties and is involved in the downregulation of epithelial markers and upregulation of mesenchymal markers [5, 6]. Vimentin is regarded as a sign of cell epithelial to mesenchymal conversion and seems to be one of the best indicators of EMT in tumorigenesis [7, 8]. Vimentin plays a vital role in the progression and prognosis of cancer via the EMT and the corresponding signaling pathways, which contributes to the tumorigenesis, metastasis, invasion, and therapeutic resistance of various tumors [9, 10]. Accumulating evidences have demonstrated that vimentin overexpression stimulates the metastasis and invasion of CRC [11–13]. However, its prognostic significance remains unclarified. A previous study suggested that vimentin could be a promising predictive marker for patients with stage III CRC [14], whereas a recent study indicated that vimentin was of no prognostic value for these patients [15].

To the best of our knowledge, so far there has been no systematic review on the prognostic significance of vimentin expression in CRC. Therefore, we conducted a study based on a meta-analysis and TCGA database to estimate the relations between vimentin and prognosis and progression in CRC.

## 2. Materials and Methods

### 2.1. Meta-Analysis

#### 2.1.1. Search Strategy

Following the Preferred Reporting Items for Systematic Reviews and Meta-analysis (PRISMA) statements checklist, PubMed, Embase, ClinicalKey, CNKI, VIP, and WanFang databases were searched until Apr. 2018[16]. The search terms were as follows: (Vimentin or vim or vmt or vm or hel113 or ctrct30) and (colorectal or colon or rectum or colorectum) and (cancer or carcinoma or adenocarcinoma or tumor or neoplasm). No restrictions were placed on language. References of the retrieved and review articles were also screened by hand.

#### 2.1.2. Selection Criteria

The included studies had to meet the following criteria: (1) patients with a pathological diagnosis of stage I to IV CRC who underwent radical surgery, (2) studies detected the level of vimentin protein or vimentin mRNA in the CRC tissues by immunohistochemistry (IHC) or real-time reverse transcription-polymerase chain reaction (qRT-PCR), (3) studies investigated the association of vimentin expression with the overall survival (OS), disease-free survival (DFS) or clinicopathological features such as age, gender, tumor size, differentiation, TNM stage, lymph node metastasis, and distant metastasis, (4) the survival data may be directly or indirectly obtained, and (5) when an author had several studies on the same patient population, only the most recent or largest sample article was included. The exclusion criteria in our meta-analysis included (1) titles, abstracts, systematic review, meta-analysis, case reports, letters, and conference data, (2) duplicated study, (3) studies used animals, cell lines, or others but not tumor tissues, (4) no effective data to estimate HR with its 95%CI, (5) studies combined vimentin with other markers to evaluate its clinical significance in CRC, or (6) a study with low quality.

#### 2.1.3. Data Extraction

Data were extracted independently by two authors, and the inconsistent opinions were adjudicated by a third author. The information collected from each study are as follows: the first authors' last name, year of publication, countries, the study design, the number of patients, cancer site, the stage of cancer, the follow-up time, treatment, technology of detection, the value of cut-off, the type of survival analysis, and HR with 95%CI. Moreover, clinicopathological parameters were collected, including age, gender, tumor size, tumor site, serum CEA (carcinoembryonic antigen) level, differentiation, lymph node metastasis, distant metastasis, recurrence metastasis, lymphovascular invasion, venous invasion, TNM stages, T stage, and N stage.

#### 2.1.4. Quality Assessment

The quality of each study was evaluated by the Newcastle-Ottawa Scale (NOS), which is a 9-star system containing the following three dimensions: the selection of cohorts, the comparability of cohorts, and the ascertainment of outcomes [17]. A study with 7-9 scores was classified as a high-quality study, whereas those with scores of 4–6 and 0-3 are moderate- and low-quality studies, respectively [18].

#### 2.1.5. Statistical Analysis

The data analyses were performed using Comprehensive Meta-Analysis Software, v. 2.0 (CMA, Biostat, Englewood, NJ, USA). The prognostic value of vimentin expression in the CRC patients was estimated by summary HRs with 95%CIs. The HRs and 95%CIs were obtained directly from the univariate or multivariate survival analysis and indirectly from Kaplan–Meier survival curves as reported by Parmar [19]. Pooled ORs and corresponding 95%CIs were calculated to evaluate the relations between vimentin expression and the clinicopathological features, including age, gender, tumor size, tumor site, serum CEA level, differentiation, lymph node metastasis, distant metastasis, recurrence metastasis, lymphovascular invasion, venous invasion, TNM stages, T stage, and N stage. Heterogeneity was evaluated among studies by calculating the *Q*-statistic and *I*^2^ value. A significant heterogeneity was present among studies if a* p* value of <0.10 for the *Q*-test, the *I*^2^ value describes the percentage of variation across studies that are due to heterogeneity rather than chance, while an *I*^2^ of 0% indicates no observed heterogeneity, with 25% regarded as low, 50% as moderate, and 75% as high [20]. Furthermore, the random-effects model was used to provide more conservative pooled estimates [21]. Publication bias was assessed by constructing the funnel plots (there was no publication bias if the funnel plot was symmetric) and quantified using Begg's test [22] and Egger's test [23], in which a* p*-value<0.05 indicated the existence of potential publication bias. A sensitivity analysis was also performed to assess whether the combined estimates could have been markedly influenced by a single study, in which each study was omitted one by one and the analysis was repeated based on the remaining studies.

### 2.2. Analysis of the Cancer Genome Atlas (TCGA) Database

We downloaded the data of 344 CRC cases on age, gender, race, tumor stage, survival information, and vimentin expression. Based on the median of vimentin expression, all cases were divided into high-expression (n=165) and low-expression groups (n=179). Survival rates were estimated by multivariate analysis. Cox regression analysis was used to perform the multivariate analysis, in which the confounding factors including age, gender, tumor stage, and race were adjusted. The results were considered statistically significant if* p* < 0.05.

## 3. Results

### 3.1. Meta-Analysis

#### 3.1.1. Literature Search

**Figure 1:**
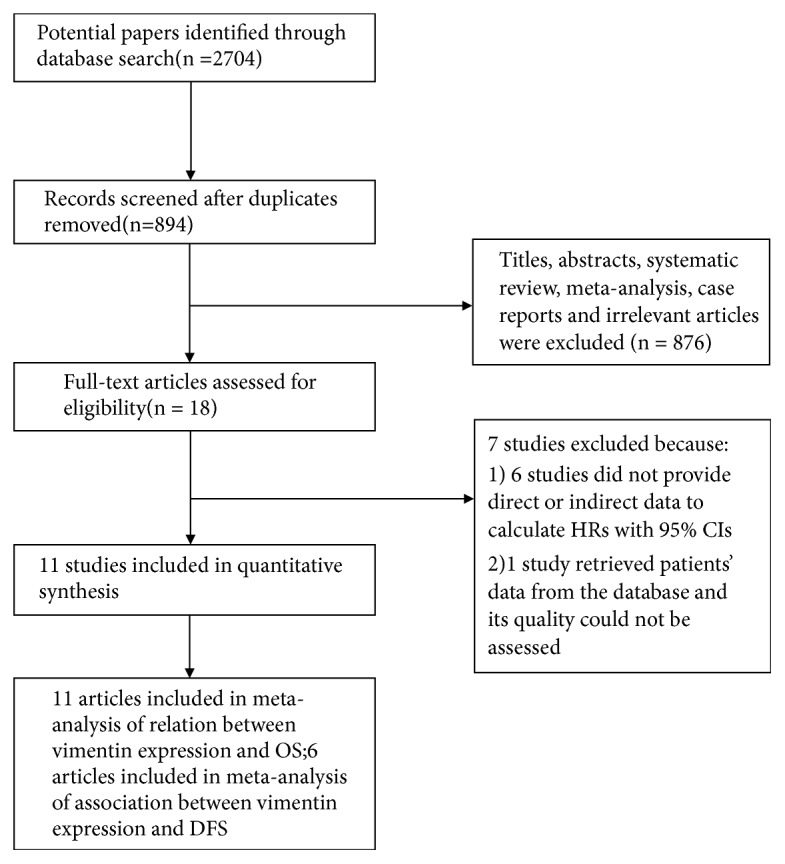
**Flow diagram of the study selection in this meta-analysis**. NOS: Newcastle-Ottawa-Scale; OS: overall survival; DFS: disease-free survival.

The flow diagram of the literature search is shown in Figure 1. We searched 2704 titles or abstracts until the latest date of Apr. 2018, of which 18 articles were related to our research purpose. Finally, a total of 11 studies were included in this meta-analysis, and the main reasons for removing 7 studies from the remaining articles were as follows: 6 studies provided insufficient data for calculating HRs with 95%CIs [11, 13, 24–27], and 1 study retrieved patient data from the prospectively maintained hepato-pancreato-biliary database and its quality could not be assessed [28].

#### 3.1.2. Study Characteristics and Quality Assessment

The main characteristics of the included studies are summarized in Tables 1 and 2. The type of study design was cohort study with 1969 cases. 7 studies were conducted in China [29–35], 2 studies were conducted in Japan [12, 14], and 2 studies were conducted in Korea [15, 36]. All of the included studies evaluated the correlation between vimentin expression and OS. Of these studies, 6 studies also assessed the association of vimentin expression with DFS [12, 14, 15, 29, 31, 34]. The quality scores of studies ranged from 7 to 9. Therefore, all of the included studies were high-quality studies (studies with a score*⩾*7), as shown in Table 1.

#### 3.1.3. Association of Vimentin Expression and OS

**Figure 2:**
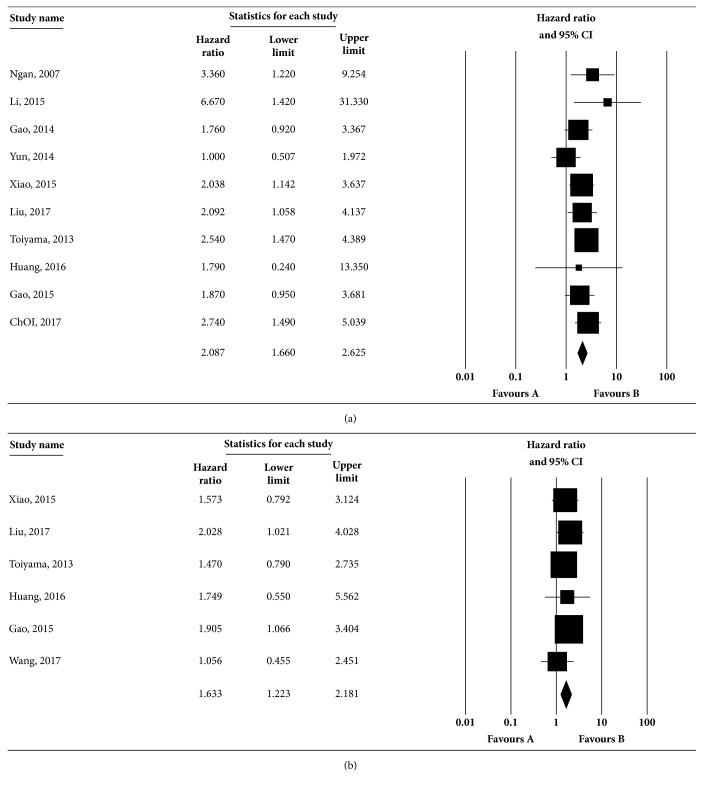
**Association between vimentin expression and OS rate in CRC by univariate (a) and multivariate (b) analyses**. Studies were combined using the random-effects model. (a) The pooled HR for OS was 2.087 (95% CI: 1.660-2.625;* p* for heterogeneity = 0.421, I^2^= 1.966%). (b) The pooled HR for OS was 1.633 (95% CI: 1.223-2.181;* p* for heterogeneity =0.874, I^2^=0%). The square boxes indicate study-specific estimates. The size of each box reflects the study's weight in the analysis, and the horizontal lines represent 95% CIs. The diamond represents the pooled HRs and 95% CI. The* p* value < 0.1 indicated the existence of heterogeneity among studies.

A total of 11 studies investigated the significance of vimentin expression in the OS of CRC, of which 10 studies performed the univariate analysis, and 6 studies performed the multivariate analysis. In the univariate analysis, the heterogeneity was not statistically significant (Q=9.180, I^2^=1.966%,* p*=0.421), and the results of the pooled HR showed that positive vimentin expression predicted a poor OS (HR: 2.087, 95%CI: 1.660-2.625) based on the random-effects model, as shown in Figure 2(a). Moreover, the multivariate analysis indicated the relation between positive vimentin expression and an unfavorable OS (HR: 1.633, 95%CI: 1.223-2.181, Figure 2(b)) by the random-effects model. There was not significant heterogeneity among the studies (Q=1.818, I^2^=0%,* p*=0.874). In sensitivity analyses, no great fluctuation was observed in the pooled results when one study was ruled out in univariate or multivariate analyses (Fig S1A and B), which suggested that the results of this meta-analysis were reliable. Begg and Egger tests were conducted to evaluate the publication bias of the included studies. No evident publication bias was detected based on the symmetric distribution of funnel plot and* P *values in Begg (*p*=0.655 in univariate analysis, Fig S2A,* p*=0.851 in multivariate analysis, Fig S2B) and Egger tests (*p*=0.454 in univariate analysis, Fig S2A*, p*=0.562 in multivariate analysis, Fig S2B). The subgroup analyses were also conducted to verify the above findings, and the detailed results are summarized in Table 3.

#### 3.1.4. Association of Vimentin Expression and DFS

**Figure 3:**
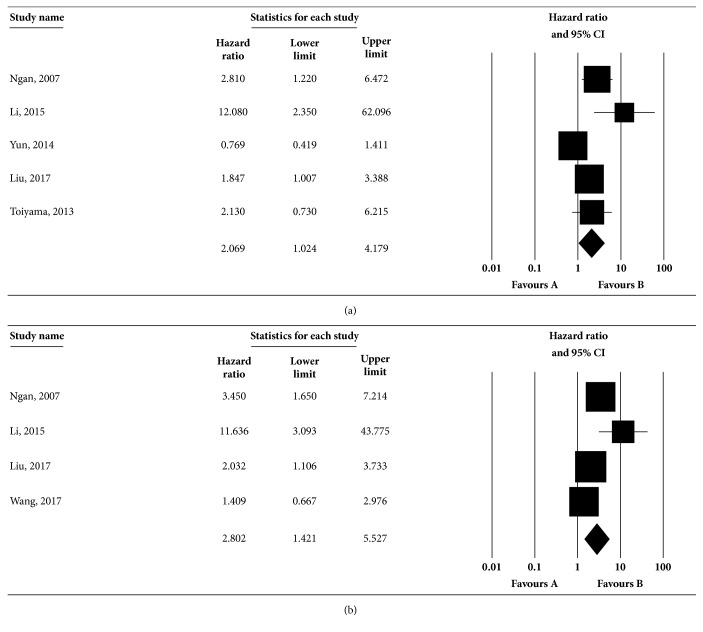
**Association between vimentin expression and DFS rate in CRC by univariate (a) and multivariate (b) analyses**. Studies were combined using the random-effects model. (a) The pooled HR for DFS was 2.069 (95% CI: 1.024-4.179;* p* for heterogeneity =0.008, I^2^=70.734%). (b) The pooled HR for DFS was 2.802 (95% CI: 1.421-5.527;* p* for heterogeneity =0.035, I^2^=65.133%). The square boxes indicate study-specific estimates. The size of each box reflects the study's weight in the analysis, and the horizontal lines represent 95% CIs. The diamond represents the pooled HRs and 95% CI. The* p* value < 0.1 indicated the existence of heterogeneity among studies.

Six papers provided data on the effect of vimentin expression on the DFS of CRC. Among these studies, 5 studies conducted the univariate analysis, and 4 studies conducted the multivariate analysis. The univariate analysis showed that the combined HR was 2.069 (95%CI: 1.024-4.179, Figure 3(a)) via the random-effects model with substantial heterogeneity (Q=13.668, I^2^=70.734%,* p*=0.008); the multivariate analysis indicated that the estimated effect was HR 2.802 (95%CI: 1.421-5.527, Figure 3(b)) based on the random-effects model with potential heterogeneity (Q=8.604, I^2^=65.133%,* p*=0.035). The result of sensitivity test in univariate analysis showed that there was no statistical significance in the pooled HRs of the remaining studies by omission of Ngan, 2007, Li, 2015, Liu, 2017 and Toiyama, 2013 (Fig S1C), which were not consistent with the combined estimates. Therefore, the four studies might be the sources of significant heterogeneity. However, excluding any single study did not affect the result of DFS in multivariate analysis (Fig S1D), which needs further discussion. Publication bias was estimated by Begg and Egger test. There was no indication of publication bias based on the symmetric distribution of funnel plot and* P* values in Begg (*p*=0.142 in univariate analysis, Fig S2C,* p*=0.497 in multivariate analysis, Fig S2D) and Egger tests (*p*=0.119 in univariate analysis, Fig S2C,* p*=0.217 in multivariate analysis, Fig S2D).

#### 3.1.5. Correlations between Vimentin and Clinicopathological Characteristics

**Figure 4:**
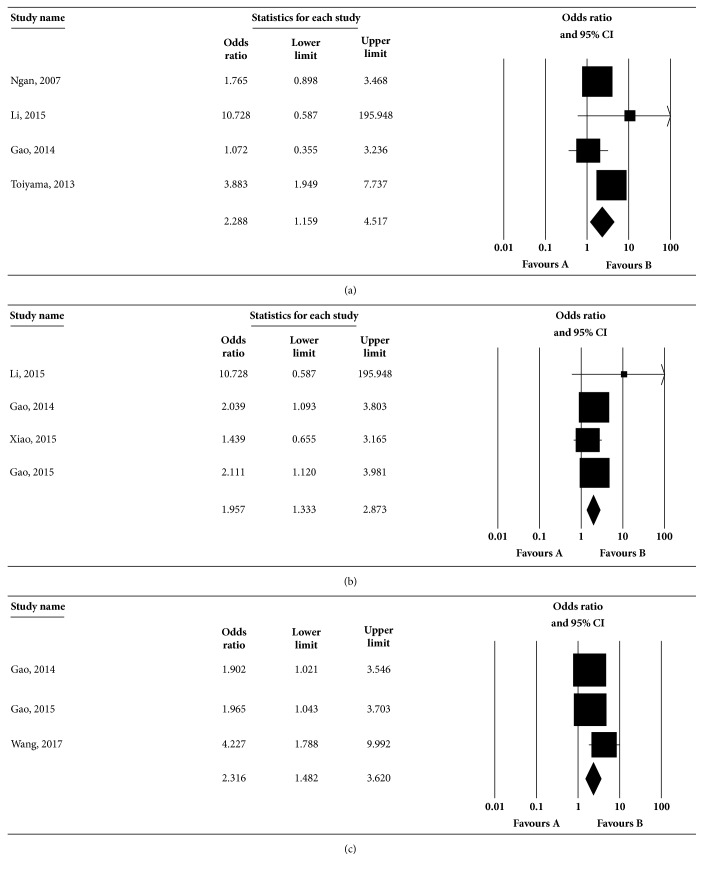
**Correlations between vimentin expression and clinicopathological characteristics.** Studies were combined using the random-effects model. (a) The pooled OR for lymph node metastasis was 2.288 (95% CI: 1.159-4.517;* p* for heterogeneity =0.126, I^2^=47.52%). (b) The pooled OR for TNM stages was 1.957 (95% CI: 1.333-2.873;* p* for heterogeneity =0.578, I^2^=0%). (c) The pooled OR for N stage was 2.316 (95% CI: 1.482-3.620;* p* for heterogeneity =0.284, I^2^=20.477%). The square boxes indicate study-specific estimates. The size of each box reflects the study's weight in the analysis, and the horizontal lines represent 95% CIs. The diamond represents the pooled ORs and 95% CI. The* p* value < 0.1 indicated the existence of heterogeneity among studies.

Ten studies were included to estimate the association between vimentin expression and clinicopathological characteristics in CRC.  Table 4 showed the combined ORs of vimentin in various parameters. The summary results suggested that positive vimentin expression was related to lymph node metastasis (OR: 2.288, 95%CI: 1.159-4.517, Figure 4(a)), TNM stages (OR: 1.957, 95%CI: 1.333-2.873, Figure 4(b)), and N stage (OR: 2.316, 95%CI: 1.482-3.620, Figure 4(c)). No significant correlation was observed between vimentin and other clinicopathological characteristics (*p*>0.05). The results of the sensitivity analysis indicated that the summary results were not influenced by excluding any one study in each clinicopathological characteristic (data not shown). There was no significant publication bias in the majority of the clinicopathological characteristics except venous invasion and N stage (*p*<0.05, data not shown).

### 3.2. Analysis of the Cancer Genome Atlas (TCGA) Database

**Figure 5:**
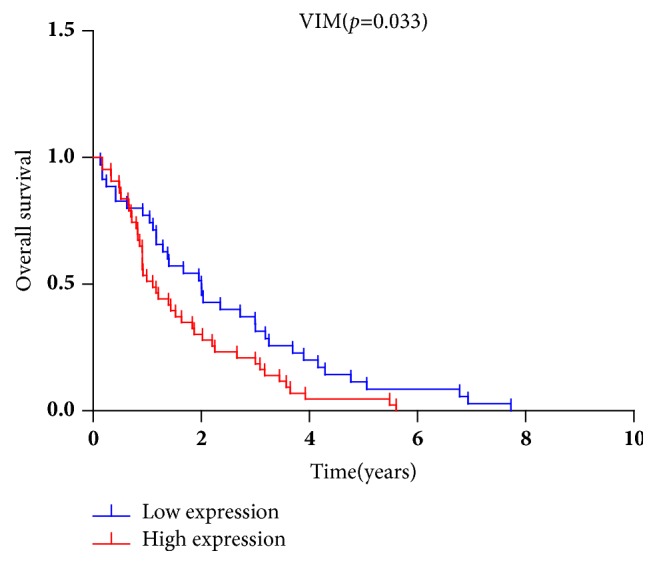
**Increased vimentin expression was associated with reduced OS in CRC.** The data on survival and vimentin expression downloaded from TCGA database indicated 165 cases with vimentin upregulation and 179 cases with vimentin downregulation. Survival rates were estimated using Cox regression analysis. The* p* value <0.05 was considered statistically significant.

The association between vimentin expression and prognosis of CRC was also evaluated from TCGA data. The result suggested that high vimentin expression indicated a shorter OS compared with low-expression group (*p*=0.033, Figure 5).

## 4. Discussion

A total of 11 cohort studies on the relation between vimentin expression and the prognosis of CRC were included in this review. To our knowledge, this report is the first meta-analysis combined with TCGA database to evaluate the value of vimentin in predicting the progression and prognosis of CRC. The results of meta-analysis suggested that positive vimentin expression predicted a poorer OS in both univariate and multivariate analyses. In the univariate analysis of DFS, the combined HR indicated that the association of positive vimentin expression with the shorter survival in CRC. Moreover, in the multivariate analysis of DFS, in which confounding factors are adjusted, we found that vimentin could be a significant prognostic factor. The potential heterogeneities existed in the two analyses of DFS. The main reasons are as follows: (1) excluding Ngan, 2007 [14], Li, 2015 [29], Liu, 2017 [31], and Toiyama, 2013 [12], influenced the pooled HR of DFS in univariate analysis; (2) the results of publication bias showed that the funnel plots were not symmetric in univariate and multivariate analyses, which may be the sources of significant heterogeneities among studies; (3) the survival data were obtained by calculation based on survival curves in three studies [12, 14, 29], which may result in an inaccurate HR of DFS. The results of the current meta-analysis also indicated that upregulated vimentin correlated well with lymph node metastasis, advanced TNM stages, and N stage. Moreover, substantial heterogeneities were observed in differentiation, distant metastasis, recurrence, and lymphovascular invasion, which may depend on the differences in country, cancer stage, and sample size among the included studies. The analysis from TCGA database indicated that elevated vimentin expression predicted a shorter OS.

Vimentin is a major component of the intermediate filament (IF) family and is involved in maintaining the cellular integrity and stability [37]. Increasing studies investigated the prognostic roles of vimentin expression and its clinicopathological significance in cancer [38–41]. However, the results of the published studies were inconsistent. A recent study indicated that vimentin overexpression in the invasive front of CRC significantly correlated with poor OS (*p*=0.028) [36], which is similar to our findings. Besides, a novel study based on computational modeling also supported this conclusion and identified vimentin as a valuable biomarker for CRC [42]. However, Yun et al. revealed that vimentin failed to indicate a significant association with prognosis in CRC [15]. The contradictions between the published studies may result from the differences in sample size, CRC stage, and the study design. Moreover, the prognosis-indicative role of vimentin was shown in other cancers and diseases. Nakashima et al. found that vimentin expression was markedly upregulated in micropapillary components of lung adenocarcinomas and it predicted adverse clinical outcome [43]. Tian et al. investigated the prognostic role of E-cadherin and vimentin expression in various subtypes of soft tissue leiomyosarcomas (LMS). They suggested that the patients with the gain of E-cadherin and loss of vimentin expression represented favorable trend of survival. Furthermore, the two markers might serve as good biomarkers of the LMS clinical outcome [44]. Our findings also showed that the overexpression of vimentin was associated with lymph node metastasis, advanced TNM stages, and N stage, whereas no significant relation was observed between upregulated vimentin and age, tumor size, gender, tumor site, serum CEA level, differentiation, distant metastasis, recurrence metastasis, lymphovascular invasion, venous invasion, and T stage. The increasing evidences suggested that high vimentin expression correlated well with the clinicopathological characteristics in other cancers such as cholangiocarcinoma (CCA), lung cancer, and liver cancer [45–47]. The findings were consistent with the results of our study. Hence, vimentin expression played a crucial role in the progression and prognosis of CRC.

Additionally, other markers could influence the progression and prognosis of cancer through regulating vimentin expression. A recent study reported that vimentin overexpression and the EMT were induced by PLAGL2 via Wnt/*β*-catenin signaling pathway in CRC, which stimulated the migration and invasion of tumor cells and may validate our findings that vimentin was related to lymph node metastasis in CRC [48]. The EMT of CRC were inhibited by loss of BMI-1 in inflammatory microenvironment through TLR4/MD-2/MyD88-mediated NF-*κ*B signaling, which was beneficial to the prognosis of CRC [49]. Moreover, the expression of EMT-associated genes could be regulated by microRNAs. A recently published study indicated that miR-194 significantly upregulated vimentin expression in CRC, which resulted in cell migration and promoted the development of CRC [50]. MiR-375 inhibited the invasion and metastasis of CRC via targeting SP1 and regulating EMT-associated genes [51]. The EMT and metastasis in CRC were also suppressed by long noncoding RNA LINC01133 directly binding to SRSF6 [52]. In addition, the EMT of CRC cells was regulated by the renin angiotensin system, where vimentin expression was reduced by the blocker of renin angiotensin system peptide ANG II type 1 receptor (AT1R) and thus inhibited the metastasis and invasion of CRC [53]. Because of the significantly prognostic role of vimentin, researchers focused on its effect on the treatment of cancer. Lahat et al. showed that vimentin was a novel anticancer therapeutic target by mice xenograft studies [54]. Subsequently, cancer cell biologists turned their attention to reprogramming cancer stem cells to normal stem cells. Hugwil reported that a human monoclonal antibody, CLN-IgG, recognized vimentin expressing on the cell surface of the malignant tumor to reprogram cancer stem cells to normal organogenesis and thereby suppressed the progression of cancer [55]. In recent years, most anticancer products and drugs exerted their functions effectively by acting on multiple anticancer molecular targets. A recent study showed that cyclometalated gold (III) complexes realized its anticancer effect by the specific engagement with multiple cellular targets including vimentin [56]. Some anticancer drugs could inhibit migration and invasion of tumor cells by regulating EMT-associated genes expression [57, 58].

There are limited studies on the role of vimentin in the progression and prognosis of CRC patients and the results are inconsistent among studies. Thus, we first conducted a study with meta-analysis and TCGA database to investigate the value of vimentin in predicting the progression and prognosis of CRC. Compared with a single study with small sample size, a meta-analysis can provide a more stable result and make a more convincing conclusion because it summarizes the single sample data and combines all the existing evidences. Furthermore, we applied TCGA database to verify the prognostic value of vimentin in CRC, which includes the complete and updated data and thus could make the conclusions more reliable.

Similar to all studies, the present study has several limitations. First, the number of the included studies and sample size are smaller (a total of 11 included articles with 1969 cases). When the number of the studies is smaller than 10 in subgroup analysis, the power of publication bias test is declined and the combined results are unstable [59]. Thus, prospective studies with large sample sizes are needed to confirm the value of vimentin in predicting the progression and prognosis of CRC. Second, six studies did not directly provide the survival data, and the HRs of OS and DFS were obtained by calculation according to survival curves, which may cause an inaccurate pooled HR [14, 29, 30, 33, 35, 36]. Moreover, we could not perform subgroup analysis in assessing the relations between vimentin and clinicopathological characteristics due to the smaller included studies. Finally, the positive vimentin expression was defined based on cut-off value in each study, and the values were inconsistent among the included studies. Therefore, the heterogeneities may exist among the studies, which may weaken the reliability of the combined results and influence the conclusions.

This is the first study with meta-analysis and TCGA database to demonstrate that positive vimentin expression predicted a poor OS and DFS in both univariate and multivariate analyses. Additionally, upregulated vimentin was related to lymph node metastasis, advanced TNM stages, and N stage. In summary, high vimentin expression contributes to the progression and poor outcome of CRC patients. Vimentin may be a promising biomarker for survival prediction and a potential target for the treatment strategies in patients with CRC. In the future, our findings should be confirmed by more well-designed cohort or experimental studies.

## Figures and Tables

**Table 1:** Characteristics of the included studies for the overall survival (OS) analysis.

Study	Country and design	Case (N)	Cancer site	Stage	Follow-up (M)	Treatment	Technique	Cut off	Overall Survival HR (95% CI)		NOS
									Univariate analysis	Multivariate analysis	
Ngan, 2007	Japan; Cohort	142	CRC	II,III	66	resection, chemotherapy^$^	IHC	>8.8%	3.360(1.220-9.250)^∧^	-^&^	7
Li, 2015	China; Cohort	41	Colon cancer	I-IV	64^#^	resection	IHC	scores≥2	6.670(1.420-31.380)^∧^	-	8
Gao, 2014	China; Cohort	194	CRC	I-IV	52^*∗*^	resection	IHC	>10%	1.760(0.920-3.370)^∧^	-	7
Yun, 2014	Korea; Cohort	409	CRC	III	80^#^	resection	IHC	scores≥1	1.000(0.507–1.975)	-	8
Xiao, 2015	China; Cohort	105	CRC	I-IV	81^#^	resection	IHC	Scores 2–3	2.038(1.142–3.795)	1.573(0.792–2.133)	8
Liu, 2017	China; Cohort	203	CRC	II	80^#^	resection	IHC	scores≥6	2.092(1.058–4.135)	2.028(1.021–4.029)	8
Toiyama, 2013	Japan; Cohort	181	CRC	I-IV	40^*∗*^	resection	qRT-PCR and IHC	scores>2	2.540(1.470-4.380)	1.470(0.790–2.750)	8
Huang, 2017	China; Cohort	117	Colon cancer	II	47^*∗*^	resection, chemotherapy^$^	IHC	Scores 2-4	1.790(0.240-13.320)^∧^	1.749(0.550–5.847)	7
Gao, 2015	China; Cohort	189	CRC	I-IV	52^*∗*^	resection	IHC	>10%	1.870(0.950-3.690)^∧^	1.905(1.066-3.407)	7
Wang, 2017	China; Cohort	102	CRC	I-III	56^*∗*^	resection	IHC	scores>3	-	1.056(0.455–2.451)	9
ChOI, 2017	Korea; Cohort	286	CRC	I-IV	53	resection, chemotherapy, and radiation therapy^$^	IHC	>5%	2.740(1.490-5.060)^∧^	-	7

CRC, colorectal cancer; qRT-PCR, real-time reverse transcription-polymerase chain reaction; IHC, immunohistochemistry; NOS, Newcastle-Ottawa Scale; N, the number of cases; M, months.

^*∗*^: median follow-up time.

^#^: maximum follow-up time.

^$^: adjuvant chemotherapy or radiation therapy after surgical resection.

^&^-: not available.

^∧^: data calculated from Kaplan–Meier survival curves.

**Table 2:** Characteristics of the included studies for the disease-free survival (DFS) analysis.

Study	Country and design	Case (N)	Cancer site	Stage	Follow-up (M)	Treatment	Technique	Cut off	Disease-free Survival HR (95% CI)		NOS
									Univariate analysis	Multivariate analysis	
Ngan, 2007	Japan; Cohort	142	CRC	II,III	66	resection, chemotherapy^$^	IHC	>8.8%	2.810(1.220-6.480)^∧^	3.450(1.650–7.220)	7
Li, 2015	China; Cohort	41	Colon cancer	I-IV	64^#^	resection	IHC	scores≥2	12.080(2.350-62.000)^∧^	11.636(3.093-43.770)	8
Yun, 2014	Korea; Cohort	409	CRC	III	80^#^	resection	IHC	scores≥1	0.769(0.419–1.413)	-^&^	8
Liu, 2017	China; Cohort	203	CRC	II	80^#^	resection	IHC	scores≥6	1.847(1.007–3.386)	2.032(1.106–3.734)	8
Toiyama, 2013	Japan; Cohort	181	CRC	I-IV	40^*∗*^	resection	qRT-PCR and IHC	scores>2	2.130(0.730-6.170)^∧^	-	
Wang, 2017	China; Cohort	102	CRC	I-III	56^*∗*^	resection	IHC	scores>3	-	1.409(0.667–2.975)	9

CRC, colorectal cancer; IHC, immunohistochemistry; NOS, Newcastle-Ottawa Scale; N, the number of cases; M, months.

^*∗*^: median follow-up time.

^#^: maximum follow-up time.

^$^: adjuvant chemotherapy after surgical resection.

^&^-: not available.

^∧^: data calculated from Kaplan–Meier survival curves.

**Table 3:** Subgroup analysis of HR in overall survival (OS) by univariate and multivariate analyses.

Variables	Study (N)	Heterogeneity test			HR (95%CI)	p
		Q	I^2^ (%)	p		
Overall survival (U)	10	9.180	1.966	0.421	2.087(1.660-2.625)	0.000^*∗*^
Country						
China	6	2.536	0.000	0.771	2.041(1.476-2.823)	0.000^*∗*^
Japan	2	0.227	0.000	0.634	2.717(1.638-4.506)	0.000^*∗*^
Korea	2	4.688	78.670	0.030	1.740(1.083-2.796)	0.022^*∗*^
Sample						
≤200	7	3.683	0.000	0.719	2.241(1.667-3.012)	0.000^*∗*^
>200	3	4.873	58.956	0.087	1.842(1.242-2.732)	0.002^*∗*^
Cancer site						
CRC	8	6.950	0.000	0.434	2.038(1.619-2.565)	0.000^*∗*^
Colon	2	1.034	3.255	0.309	4.088(1.200-13.928)	0.024^*∗*^
Year						
2014-2017	8	7.517	6.882	0.377	1.938(1.500-2.505)	0.000^*∗*^
Before 2014	2	0.227	0.000	0.634	2.706(1.672-4.378)	0.000^*∗*^
Overall survival (M)	6	1.818	0.000	0.874	1.633(1.223-2.181)	0.001^*∗*^
Country						
China	5	1.677	0.000	0.795	1.681(1.213-2.331)	0.002^*∗*^
Japan	1	-	-	-	-	-
Sample						
≤200	5	1.353	0.000	0.852	1.559(1.133-2.144)	0.006^*∗*^
>200	1	-	-	-	-	-
Cancer site						
CRC	5	1.804	0.000	0.772	1.626(1.206-2.191)	0.001^*∗*^
Colon	1	-	-	-	-	-
Year						
2014-2017	5	1.677	0.000	0.795	1.681(1.213-2.331)	0.002^*∗*^
Before 2014	1	-	-	-	-	-

N, the number of the included studies; U, univariate analysis; M, multivariate analysis; CRC, colorectal cancer.

^*∗*^: the value of *p*<0.05 indicates statistical significance.

^&^-: not available.

**Table 4:** Analysis of relationships between vimentin and clinicopathological variables in CRC.

Clinical pathological variable	Study (N)	Pooled OR (95%CI)	p value	Heterogeneity test		
				Q	I^2^ (%)	p
Age (≤60/>60)	6	0.964(0.742-1.253)	0.786	1.368	0.000	0.928
Tumor size (>5cm/≤5cm)	5	0.782(0.581-1.053)	0.105	2.582	0.000	0.630
Gender (male/female)	10	0.943(0.761-1.169)	0.594	5.800	0.000	0.760
Tumour site (Colon/Rectum)	5	0.929(0.690-1.250)	0.626	3.835	0.000	0.429
CEA level (>5ng/ml/≤5 ng/ml)	3	0.937(0.650-1.352)	0.728	1.559	0.000	0.459
Differentiation (poor/well or mod)	8	0.954(0.407-2.234)	0.914	53.357	86.881	0.000
Lymph node metastasis (present/absent)	4	2.288(1.159-4.517)	0.017^*∗*^	5.717	47.520	0.126
Distant metastasis (present/absent)	2	4.122(0.820-20.730)	0.086	2.551	60.807	0.110
Recurrence (present/absent)	2	3.726(0.215-64.509)	0.366	5.993	83.314	0.014
Lymphovascular invasion (present/absent)	4	1.244(0.736-2.103)	0.415	6.367	52.883	0.095
Venous invasion (present/absent)	3	1.180(0.220-6.328)	0.847	3.912	48.880	0.141
TNM stages (III-IV/I-II)	4	1.957(1.333-2.873)	0.001^*∗*^	1.973	0.000	0.578
T stage (T3–T4/T1–T2)	3	0.756(0.520-1.098)	0.142	1.242	0.000	0.537
N stage (N1–N2/N0)	3	2.316(1.482-3.620)	0.000^*∗*^	2.515	20.477	0.284

N, the number of the included studies; CEA, carcinoembryonic antigen; CRC, colorectal cancer.

^*∗*^: the value of *p*<0.05 indicates statistical significance.

## Supplementary Materials

Supplementary Materials The sensitivity analyses and publication bias of the included studies.
