# Supplementary material for: High Vimentin Expression Predicts a Poor Prognosis and Progression in Colorectal Cancer: A Study with Meta-Analysis and TCGA Database
Source: Biomed Res Int. 2018 May 31;2018:6387810. doi: 10.1155/2018/6387810 (PMC6000861; doi:10.1155/2018/6387810)
Supplement: Supplementary Materials — The sensitivity analyses and publication bias of the included studies. [file 6387810.f1.pdf]

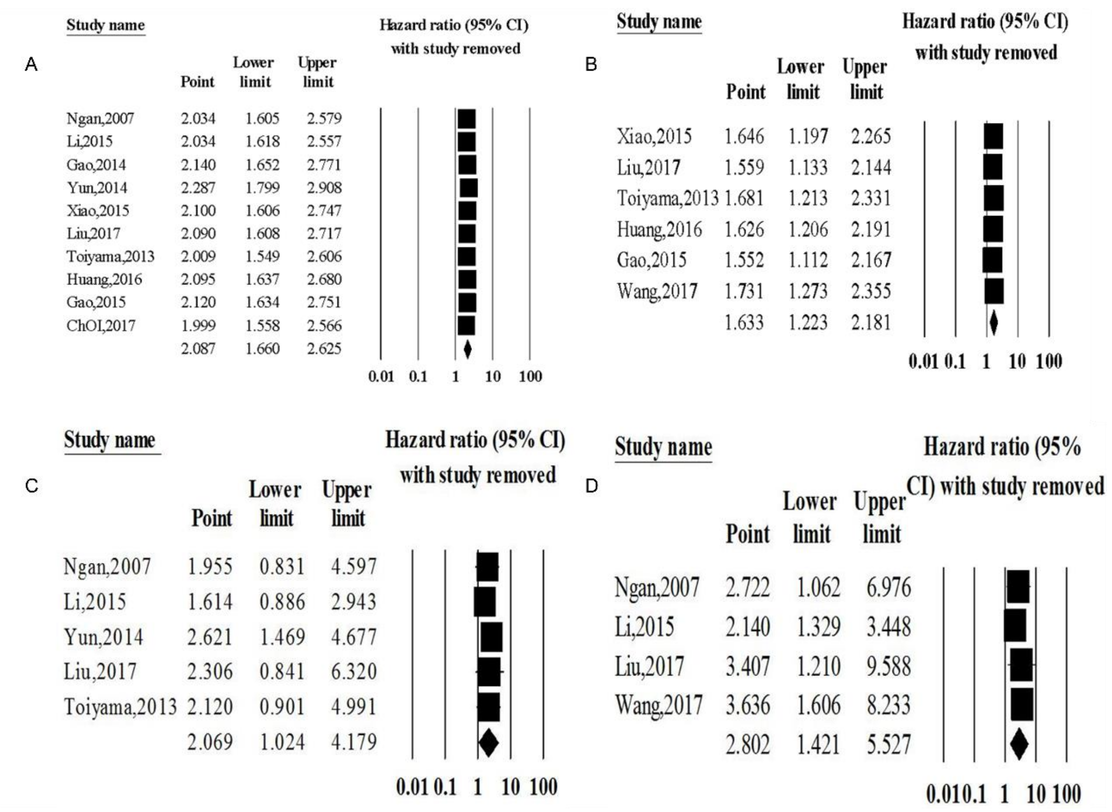

Figure S1. Sensitivity analyses of included studies. A. OS in univariate analysis. B. OS in multivariate analysis. C. DFS in univariate analysis. D. DFS in multivariate analysis. The square boxes indicate the combined estimates of the remaining studies by omission of one study. The horizontal lines represent 95% CIs. The diamond represents the pooled HRs and 95% CI.

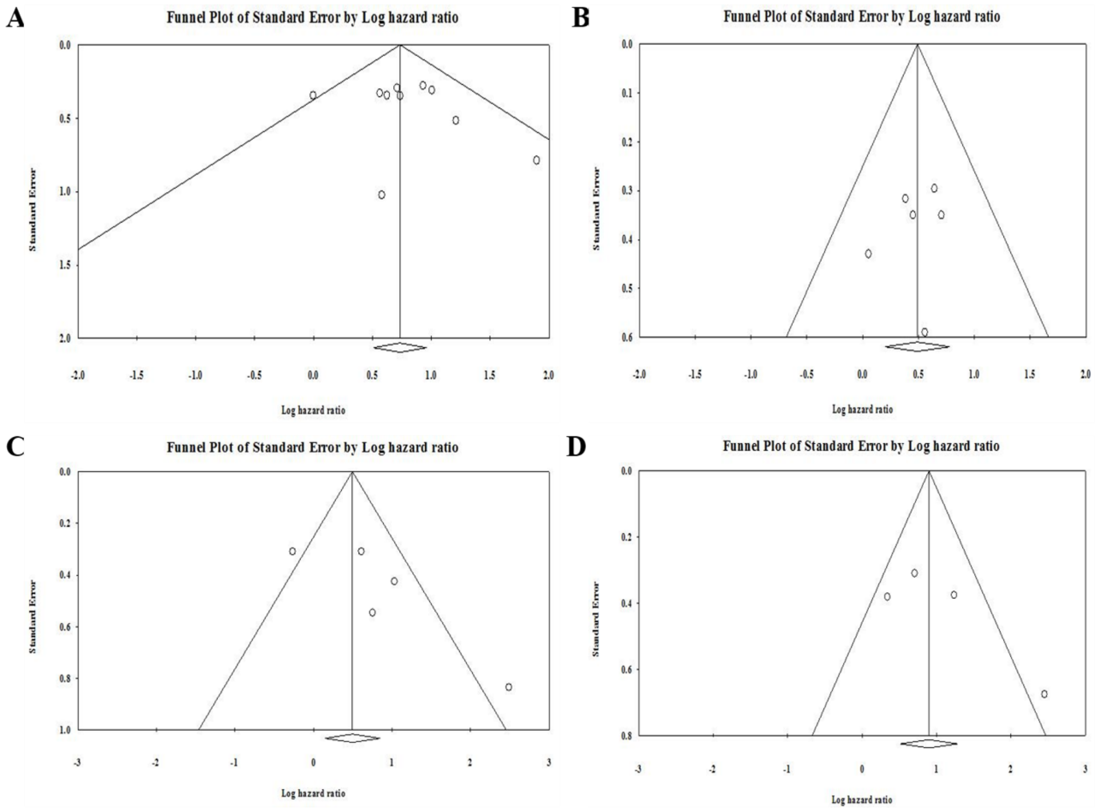

Figure S2. Funnel plots for publication bias. A. OS in univariate analysis. B. OS in multivariate analysis. C. DFS in univariate analysis. D. DFS in multivariate analysis. The circles represent each study. The vertical bars indicate the pooled log HRs. Two diagonal lines reflect 95% CIs. The symmetric funnel plot was considered no publication bias.
